# High-Dose Corticosteroid Therapy in Facial Nerve Palsy: A Retrospective Study

**DOI:** 10.7759/cureus.81949

**Published:** 2025-04-09

**Authors:** Hiroshi Hyakusoku, Noriyuki Katsumata, Meijin Nakayama

**Affiliations:** 1 Otorhinolaryngology, Yokosuka Kyosai Hospital, Yokosuka, JPN

**Keywords:** bell’s palsy, facial nerve paralysis, high-dose corticosteroid therapy, prednisolone, ramsay hunt syndrome

## Abstract

Objectives

The difference in therapeutic efficacy between an initial dosage of 200 mg and 100 mg prednisolone (PSL) with a taper for Bell’s palsy and Ramsay Hunt syndrome was retrospectively investigated.

Methods

A total of 259 patients (172 with Bell’s palsy and 87 with Ramsay Hunt syndrome) were treated with high-dose corticosteroid therapy (HDCT) with PSL (the standard HDCT: 200 mg/day for three days with a seven-day taper, or the reduced HDCT: 100 mg/day for three days with a seven-day taper) and evaluated once a month by the Yanagihara facial nerve grading system until facial nerve paralysis was cured or six months after the onset.

Results

The therapeutic efficacy of the standard HDCT was not significantly improved, compared to the reduced HDCT, in Bell’s palsy and Ramsay Hunt syndrome, and even in less than 20.0% of electroneuronography in Bell’s palsy and Ramsay Hunt syndrome.

Conclusion

HDCT with more than an initial dosage of 100 mg/day PSL with a taper for Bell’s palsy and Ramsay Hunt syndrome does not increase the therapeutic efficacy.

## Introduction

Bell’s palsy is defined as an acute onset of facial nerve paralysis of unknown origin, with the most likely cause being the reactivation of latent herpes simplex virus type I at the geniculate ganglion within the facial nerve, and it affects individuals of any age and sex [1]. An annual incidence ranges from 11.5 to 53.3 per 100,000 people [2]. In contrast, Ramsay Hunt syndrome is caused by a varicella-zoster virus infection, characterized by rapid facial nerve paralysis, often accompanied by a vesicular rash on the auricles, external ear canals, and/or vestibulocochlear dysfunction. It is estimated that 5 out of every 100,000 people develop Ramsay Hunt syndrome per year in the United States [3].

Approximately 70% of Bell’s palsy patients achieve complete recovery without intervention [4]. Since approximately 90% of these patients achieve complete recovery with corticosteroid therapy, the American Academy of Otolaryngology-Head and Neck Surgery (AAO-HNS) strongly recommends corticosteroid therapy [5-8]. Facial paralysis in Ramsay Hunt syndrome, however, tends to be more severe, with only about 30% of patients recovering spontaneously [9]. About 50%-70% of patients achieve complete recovery with a combination of corticosteroids and antiviral therapy [10,11]. Nevertheless, there are no randomized controlled trials supporting the efficacy of corticosteroid or antiviral treatments for Ramsay Hunt syndrome, and no AAO-HNS guidelines exist specifically for this condition.

The optimal dosage of corticosteroids for Bell’s palsy remains a matter of debate [8]. Therefore, the AAO-HNS proposed two regimens (standard-dose corticosteroid therapy): prednisolone (PSL) 50 mg for 10 days, or PSL 60 mg for five days with a five-day taper [8]. In 1982, Stennert [12] reported that high-dose corticosteroid therapy (HDCT), with an initial dose of 200-250 mg/day, was associated with improved outcomes. However, the study had limitations, including a small sample size and the absence of a control group. A subsequent systematic review suggested that an initial dose of 120 mg or more of PSL is significantly more effective than a 60 mg dose [13]. Nevertheless, the superiority of HDCT over standard-dose therapy remains uncertain, as no randomized controlled trials have confirmed its efficacy.

In our standard treatment protocol, we hospitalize patients with acute facial nerve paralysis, such as Bell’s palsy and Ramsay Hunt syndrome, and administer HDCT (200 mg/day of PSL for three days with a seven-day taper) as Stennert’s regimen. For elderly patients, or those with comorbid conditions like diabetes mellitus or hypertension, we use a reduced dosage of 100 mg/day of PSL for three days with a similar tapering protocol to reduce side effects, under the supervision of the responsible attending physicians.

In this study, the difference in therapeutic efficacy between the standard and reduced HDCT groups was retrospectively investigated.

## Materials and methods

Cases

This retrospective study included patients aged 18 years or older who were hospitalized between April 2007 and October 2023 at Yokosuka Kyosai Hospital, Yokosuka, Japan, with an initial diagnosis of Bell’s palsy or Ramsay Hunt syndrome within 14 days of symptom onset. Electroneuronography (ENoG) was performed between 7 and 14 days after symptom onset to predict the long-term outcome [14]. Surface electrodes were placed over the orbicularis oris muscle, and electrical stimulation was applied to the main trunk of the facial nerve. The amplitude of the maximal evoked response was recorded and compared with that of the contralateral side. The response percentage was calculated by dividing the response amplitude on the paralyzed side by the amplitude on the non-paralyzed side [15]. Only patients who received HDCT and were followed up for more than six months, or until complete recovery, were included. Patients who could not be followed for the required duration were excluded from the study.

Treatments

All patients were hospitalized and received HDCT with PSL (200 mg/day for three days with a seven-day taper, or 100 mg/day for three days with a seven-day taper by intravenous infusion). To prevent gastric ulcers during corticosteroid treatment, a proton pump inhibitor was co-administered. During hospitalization, patients with diabetes mellitus received glycemic control using a sliding scale, while patients with hypertension were administered additional antihypertensive drugs if necessary. A diagnosis of hypertension or diabetes mellitus was established if the patient had a prior diagnosis and was undergoing treatment, or if the patient presented with clinical signs of hypertension or diabetes mellitus at the time of admission and required medical intervention. Antiviral therapy was administered either intravenously or orally to patients when deemed necessary by the responsible attending physician. Additionally, adenosine triphosphate and vitamin B12 were prescribed to support nerve recovery until the facial nerve paralysis resolved, or the follow-up assessment was completed.

Outcome measures

Following HDCT during hospitalization, the severity of facial nerve paralysis was evaluated monthly. Patients were followed until either full recovery or six months after onset. Facial nerve function was assessed using the Yanagihara facial nerve grading system (YFGS) [16,17]. Recovery was defined as achieving 36 or more points on the YFGS.

Statistical analysis

All quantitative variables were analyzed using the Mann-Whitney U test, while qualitative variables were compared using the Chi-square test. Logistic regression analysis was also performed to investigate factors. Statistical analyses were performed using GraphPad Prism version 7.02 (GraphPad Software, San Diego, CA, USA) and JMP 17 Pro (SAS Institute Inc., Cary, NC, USA). For all comparisons, a p-value of less than 0.05 was considered statistically significant.

## Results

General characteristics

Of the 336 enrolled in Bell’s palsy and Ramsay Hunt syndrome, a total of 259 patients were included in this study, excluding those who had no evaluation for facial nerve paralysis by YFGS, and no six-month follow-up without complete recovery. The cohort comprised 172 Bell’s palsy and 87 Ramsay Hunt syndrome patients (27 patients had three symptoms: facial paralysis, a painful red rash on the auricles and/or external ear canals, and vestibulocochlear dysfunction; 19 patients had two symptoms: facial paralysis and a painful red rash on the auricles and/or external ear canals; 41 patients had two symptoms: facial paralysis and vestibulocochlear dysfunction). The mean age of the cohort was 58.3 years (range 19-92), with 125 cases of right-sided and 134 cases of left-sided facial paralysis. Among the Bell’s palsy patients, 142 received HDCT with an initial dosage of 200 mg PSL, while 30 received 100 mg PSL. Among the Ramsay Hunt syndrome patients, 71 received 200 mg PSL and 16 received 100 mg PSL.

The distributions of ENoG values are shown in Table 1. One patient who developed Ramsay Hunt syndrome did not undergo ENoG. The baseline characteristics are shown in Tables 2-3. In both Bell’s palsy and Ramsay Hunt syndrome, the 100 mg PSL dosage group was significantly older than the 200 mg PSL dosage group. Additionally, in Bell’s palsy, the 100 mg PSL dosage group had a significantly higher prevalence of hypertension and diabetes mellitus, while in Ramsay Hunt syndrome, the 100 mg PSL dosage group had a significantly higher prevalence of hypertension.

**Table 1:** Distribution of ENoG values. ENoG, Electroneurography; PSL, Prednisolone

ENoG	< 10%	10% ≤ 20%	20% ≤ 40%	40% ≤
Bell’s palsy				
PSL 100 mg	4	7	7	12
PSL 200 mg	10	21	39	72
Ramsay Hunt syndrome				
PSL 100 mg	0	3	7	6
PSL 200 mg	9	12	19	30
All				
PSL 100 mg	4	10	14	18
PSL 200 mg	19	33	58	102

**Table 2:** General characteristics of Bell’s palsy. **p < 0.01, ***p < 0.001 Values presented in mean ± SD or n DM, Diabetes mellitus; ENoG, Electroneurography; HT, Hypertension; YFGS, Yanagihara facial nerve grading system; PSL, Prednisolone

Variables	PSL 200 mg (N = 142)	PSL 100 mg (N = 30)	p-value
Age (median, range)	60 (19-85)	78.5 (37-87)	<0.001***
Sex (male/female)	76/66	12/18	0.228
Side of palsy (right/left)	66/76	15/15	0.841
YFGS at the first visit	10.2 ± 6.4	10.1 ± 5.8	0.884
0-10	84 (59.2%)	17 (56.7%)	0.561
12-18	41 (28.9%)	11 (36.7%)	-
20-40	17 (12.0%)	2 (6.7%)	-
ENoG (%)	41.6 ± 24.1	35.2 ± 24.3	0.202
HT	31 (21.9%)	18 (60.0%)	<0.001***
DM	28 (19.7%)	15 (50.0%)	0.001**
Time from onset to treatment (day)	5.1 ± 2.9	5.2 ± 2.9	0.861
Antivirals prescription	98 (69.0%)	20 (66.7%)	0.830

**Table 3:** General characteristics of Ramsay Hunt syndrome. **p < 0.01, ***p < 0.001 Values presented in mean ± SD or n DM, Diabetes mellitus; ENoG, Electroneurography; HT, Hypertension; YFGS, Yanagihara facial nerve grading system; PSL, Prednisolone

Variables	PSL 200 mg (N = 71)	PSL 100 mg (N = 16)	p-value
Age (median, range)	55 (21-82)	72 (46-92)	<0.001***
Sex (male/female)	40/31	7/9	0.414
Side of palsy (right/left)	37/37	7/9	0.785
YFGS at the first visit	10.0 ± 7.4	9.8 ± 10.4	0.453
0-10	42 (59.2%)	11 (68.8%)	0.776
12-18	17 (23.9%)	3 (18.8%)	-
20-40	12 (16.9%)	2 (12.5%)	-
ENoG (%)	38.2 ± 25.2	40.4 ± 19.1	0.540
HT	16 (22.5%)	10 (62.5%)	0.005**
DM	6 (8.5%)	4 (25.0%)	0.082
Time from onset to treatment (day)	4.3 ± 2.6	4.9 ± 2.5	0.243
Antivirals prescription	64 (90.1%)	13 (81.3%)	0.383

Comparison of YFGS and recovery rate between the 100 mg and 200 mg PSL dosage groups

Bell’s Palsy

**Figure 1:**
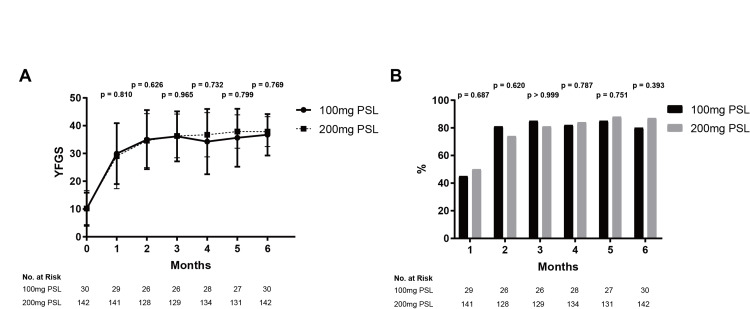
Comparison of therapeutic efficacy for Bell’s palsy patients between the 100 mg and 200 mg PSL dosage groups A) YFGS, B) Recovery rates Both YFGS and recovery rates were not significantly different at any time point between the 100 mg and 200 mg PSL dosage groups; bars denote SD YFGS, Yanagihara facial nerve grading system; PSL, Prednisolone

In the 100 mg PSL group, the mean YFGS scores were 29.9, 34.2, 36.2, 35.7, 37.1, and 36.7 points at one, two, three, four, five, and six months from hospitalization, respectively. In the 200 mg PSL group, the mean YFGS scores were 29.0, 34.6, 36.2, 37.3, 37.8, and 37.9 points at the same time points (Figure 1A). There were no significant differences in YFGS scores at any time point between the two dosage groups. The recovery rates in the 100 mg PSL group were 13/29 (44.8%), 21/26 (80.8%), 22/26 (84.6%), 23/28 (82.1%), 23/27 (85.2%), and 24/30 (80.0%) at one, two, three, four, five, and six months from hospitalization, respectively (Figure 1B). In the 200 mg PSL group, the recovery rates were 70/141 (49.6%), 95/128 (74.2%), 105/129 (81.4%), 112/134 (83.6%), 115/131 (87.8%), and 123/142 (86.6%) at the same time points. There were no significant differences in recovery rates between the two groups at any time point. Logistic regression analysis was then performed using the four variables (age, hypertension, diabetes mellitus, and dosage of PSL) as explanatory variables and recovery rate as the objective variable. All four factors were not significant variables influencing the recovery rate (Table 4).

**Table 4:** Logistic regression analysis to investigate factors for Bell’s palsy. CI, Confidence interval; DM, Diabetes mellitus; HT, Hypertension; OR, Odds ratio; PSL, Prednisolone; χ^2^, Chi-square

Explanatory variables	Standard error	Wald χ^2^-test	p-value	OR	95% CI
Age (over 65 years)	0.237	0.000	0.985	0.991	0.391-2.511
HT	0.286	1.133	0.287	0.544	0.177-1.669
DM	0.256	0.603	0.437	0.672	0.247-1.831
Dosage of PSL	0.289	0.775	0.379	1.662	0.536-5.150

Ramsay Hunt Syndrome

**Figure 2:**
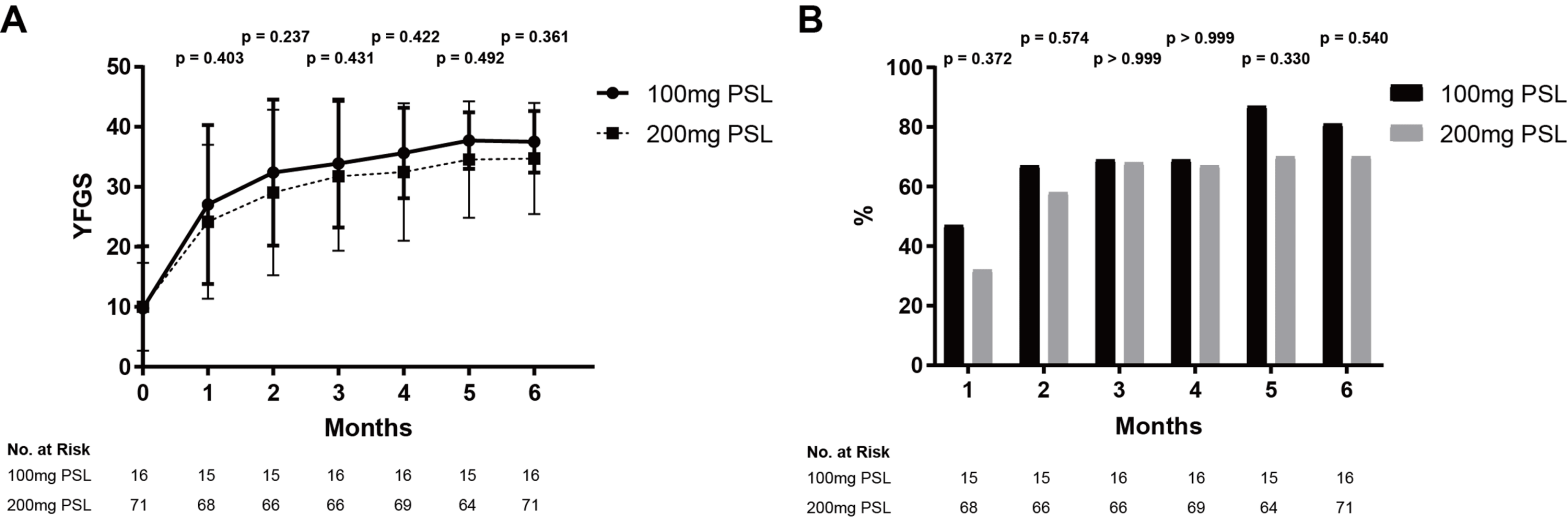
Comparison of therapeutic efficacy for Ramsay Hunt syndrome patients between the 100 mg and 200 mg PSL dosage groups. A) YFGS, B) Recovery rates Both YFGS and recovery rates were not significantly different at any time point between the 100 mg and 200 mg PSL dosage groups; bars denote SD YFGS, Yanagihara facial nerve grading system; PSL, Prednisolone

In the 100 mg PSL group, the mean YFGS scores were 27.1, 32.4, 33.9, 35.6, 37.7, and 37.5 points at one, two, three, four, five, and six months from hospitalization, respectively. In the 200 mg PSL group, the mean YFGS scores were 24.2, 29.1, 31.5, 32.5, 34.6, and 34.7 points at the same time points (Figure 2A). There were no significant differences in YFGS scores between the two dosage groups at any time point. The recovery rates in the 100 mg PSL group were 7/15 (46.7%), 10/15 (66.7%), 11/16 (68.8%), 11/16 (68.8%), 13/15 (86.7%), and 13/16 (81.3%) at one, two, three, four, five, and six months from hospitalization, respectively. In the 200 mg PSL group, the recovery rates were 22/68 (32.4%), 38/66 (57.6%), 45/66 (68.2%), 46/69 (66.7%), 45/64 (70.3%), and 50/71 (70.4%) at the same time points (Figure 2B). No significant differences in recovery rates were observed between the two dosage groups. Logistic regression analysis was then performed using the four variables (age, hypertension, diabetes mellitus, and dosage of PSL) as explanatory variables and recovery rate as the objective variable. All four factors were not significant variables influencing the recovery rate (Table 5).

**Table 5:** Logistic regression analysis to investigate factors for Ramsay Hunt syndrome. CI, Confidence interval; DM, Diabetes mellitus; HT, Hypertension; OR, Odds ratio; PSL, Prednisolone; χ^2^, Chi-square

Explanatory variables	Standard error	Wald χ^2^-test	p-value	OR	95% CI
Age (over 65 years)	0.237	0.000	0.985	0.991	0.391-2.511
HT	0.286	1.133	0.287	1.838	0.599-5.636
DM	0.256	0.603	0.437	0.672	0.247-1.831
Dosage of PSL	0.289	0.775	0.379	1.662	0.536-5.150

General characteristics of patients with <20.0% ENoG in Bell’s palsy and Ramsay Hunt syndrome

To evaluate patients with a relatively poor prognosis, we selected those with an ENoG score reduced to less than 20.0% of the contralateral value. A total of 66 patients were enrolled, including 42 with Bell’s palsy and 24 with Ramsay Hunt syndrome. Among these patients, 33 had right-sided and 33 had left-sided facial paralysis. A total of 52 patients received 200 mg PSL HDCT, and 14 received 100 mg PSL HDCT. The baseline characteristics are shown in Table 6. The 100 mg PSL dosage group was significantly older than the 200 mg PSL dosage group, and there was a significantly higher prevalence of hypertension and diabetes mellitus in the 100 mg PSL group.

**Table 6:** General characteristics of ENoG <20.0% in Bell’s palsy and Ramsay Hunt syndrome. *p < 0.05, ***p < 0.001 Values presented in mean ± SD or n DM, Diabetes Mellitus; ENoG, Electroneurography; HT, Hypertension; YFGS, Yanagihara facial nerve grading system; PSL, Prednisolone

Variables	PSL 200 mg (N = 52)	PSL 100 mg (N = 14)	p-value
Age (median, range)	55 (19-84)	77 (53-87)	<0.001***
Sex (male/female)	30/22	8/6	>0.999
Side of palsy (right/left)	25/27	8/6	0.764
YFGS at the first visit	7.3 ± 5.2	6.9 ± 4.9	0.916
0-10	40 (76.9%)	10 (71.4%)	0.596
12-18	10 (19.2%)	4 (28.6%)	-
20-40	2 (3.8%)	0 (0.0%)	-
ENoG (%)	11.5 ± 4.6	13.0 ± 5.3	0.238
Bell’s palsy/Hunt syndrome	31/21	11/3	0.227
HT	9 (17.3%)	7 (50.0%)	0.030*
DM	7 (13.5%)	9 (64.3%)	<0.001***
Time from onset to treatment (day)	4.9 ± 3.1	5.7 ± 3.2	0.390
Antivirals prescription	42 (80.8%)	10 (71.4%)	0.473

Comparison of YFGS and recovery rate with <20.0% ENoG between the 100 mg and 200 mg PSL groups

**Figure 3:**
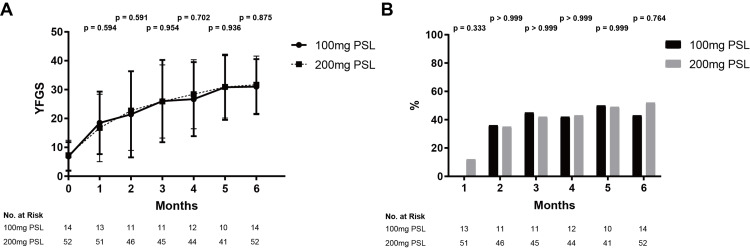
Comparison of therapeutic efficacy for patients with less than 20.0% ENoG between the 100 mg and 200 mg PSL dosage groups. A) YFGS, B) Recovery rates Both YFGS and recovery rates were not significantly different at any time point between the 100 mg and 200 mg PSL dosage groups; bars denote SD YFGS, Yanagihara facial nerve grading system; PSL, Prednisolone; ENoG, Electroneuronography

In the 100 mg PSL group, the mean YFGS scores were 18.5, 21.5, 26.0, 26.7, 30.8, and 31.0 points at one, two, three, four, five, and six months from hospitalization, respectively. In the 200 mg PSL group, the mean YFGS scores were 16.7, 22.7, 25.9, 28.4, 30.9, and 31.7 points at the same time points (Figure 3A). There were no significant differences in YFGS scores between the two groups at any time point. In the 100 mg PSL group, the recovery rates were 0/13 (0.0%), 4/11 (36.4%), 5/11 (45.5%), 5/12 (41.7%), 5/10 (50.0%), and 6/14 (42.9%) at one, two, three, four, five, and six months from hospitalization, respectively. In the 200 mg PSL group, the recovery rates were 6/51 (11.8%), 16/46 (34.8%), 19/45 (42.2%), 19/44 (43.2%), 20/41 (48.8%), and 27/52 (51.9%) at the same time points (Figure 3B). No significant differences in recovery rates were observed between the 100 mg and 200 mg PSL groups at any time point. Logistic regression analysis was then performed using the four variables (age, hypertension, diabetes mellitus, and dosage of PSL) as explanatory variables and recovery rate as the objective variable. All four factors were not significant variables influencing the recovery rate (Table 7).

**Table 7:** Logistic regression analysis to investigate factors for ENoG < 20.0% in Bell’s palsy and Ramsay Hunt syndrome. CI, Confidence interval; DM, Diabetes mellitus; HT, Hypertension; OR, Odds ratio; PSL, Prednisolone; χ^2^, Chi-square

Explanatory variables	Standard error	Wald χ^2^-test	p-value	OR	95% CI
Age (over 65 years)	0.328	0.463	0.496	0.640	0.177-2.315
HT	0.364	0.148	0.700	1.324	0.318-5.510
DM	0.354	1.176	0.278	2.156	0.538-8.644
Dosage of PSL	0.382	0.524	0.469	1.739	0.389-7.781

Complications

Elevated blood pressure and blood glucose levels are common side effects of PSL and were therefore not included in the definition of complications. Two patients (an 86-year-old male and an 83-year-old female) developed delirium, discontinued treatment, and were discharged before completing the prescribed therapy. A 55-year-old female patient discontinued hospitalization due to an anxiety disorder, but completed the treatment on an outpatient basis.

## Discussion

Comparison between the standard and reduced HDCT groups

In this study, although the reduced HDCT group was older and had a significantly higher prevalence of hypertension and diabetes mellitus, the therapeutic efficacy of the HDCT was not significantly improved compared to the reduced HDCT in Bell’s palsy and Ramsay Hunt syndrome. This conclusion was consistent even in cases with poor prognosis, where ENoG was less than 20.0% in both Bell’s palsy and Ramsay Hunt syndrome. Therefore, HDCT with more than an initial dosage of 100 mg/day PSL with a taper does not appear to enhance therapeutic efficacy for Bell’s palsy and Ramsay Hunt syndrome.

Corticosteroid therapy for Bell’s palsy

For Bell’s palsy, although corticosteroid therapy is strongly recommended by AAO-HNS, the optimal dosage remains controversial and unclear [8]. Several studies have shown mixed results, with some indicating that HDCT is more effective than a lower dosage (e.g., 60 mg/day of PSL), while others found no significant differences between these treatments [18,19]. A systematic review suggested that an initial dosage of 120 mg or more of PSL is significantly more effective than 60 mg of PSL [13]. However, Suzuki et al. reported no significant differences between an initial dose of 120-160 mg of PSL and 60 mg of PSL [20]. Based on these reports, Fujiwara et al. recommended an initial dosage of 200 mg of PSL [13]. These conflicting results suggest the need for a randomized controlled trial to definitively establish the efficacy of HDCT compared to an initial dosage of 60 mg of PSL.

Therapy for Ramsay Hunt syndrome

Ramsay Hunt syndrome is caused by infection with the varicella-zoster virus. Therefore, antiviral therapy should be performed to suppress the neuritis and neuralgia, though the efficacy of antiviral therapy for facial nerve paralysis has not been established through randomized controlled trials. Corticosteroid therapy should also be recommended for Ramsay Hunt syndrome, as it is strongly advocated for Bell’s palsy, despite the lack of verification from randomized controlled trials. Additionally, in patients with Ramsay Hunt syndrome who received both acyclovir and prednisone, Murakami et al. reported that the prognosis for facial paralysis was better in those who initiated treatment within three days of onset compared to those who initiated treatment after seven days, suggesting that combined corticosteroid and antiviral therapy may be effective for facial paralysis [11]. However, no studies have specifically compared HDCT to a 60 mg PSL regimen for Ramsay Hunt syndrome. Based on the findings of this study, a randomized controlled study is necessary to verify the effectiveness of corticosteroid therapy and to determine the optimal dosage for treatment.

Facial nerve paralysis with ENoG <20.0% in Bell’s palsy and Ramsay Hunt syndrome

In patients with a relatively poor prognosis, defined as having an ENoG of 20% or less, there were no significant differences in treatment outcomes between an initial dosage of 200 mg PSL and 100 mg PSL. These findings suggest that increasing the initial dosage of PSL beyond 100 mg does not enhance therapeutic efficacy in severe cases of Bell’s palsy or Ramsay Hunt syndrome.

Relationship with age and comorbid conditions

Although the reduced HDCT group was older and had a significantly higher prevalence of hypertension and diabetes mellitus, there were no differences in treatment outcomes between the two groups. Even when dividing these cases into recovered and unrecovered groups, no significant differences in age or comorbid conditions, such as hypertension or diabetes mellitus, were observed between the two groups. Some studies have reported that patients with comorbid conditions, such as diabetes mellitus and hypertension, have a poor prognosis for facial nerve paralysis, while others have shown no significant differences [21-24]. Diabetes mellitus causes chronic nerve ischemia and impaired nerve blood flow, which affects the process of facial nerve repair [25]. Hypertension exerts a similar effect by inducing arteriosclerosis and reducing peripheral blood flow [26]. Furthermore, age is assumed to correlate with the prognosis of facial nerve paralysis, as the prevalence of complications, such as diabetes mellitus and hypertension, increases with age. In this study, age and the prevalence of hypertension and diabetes mellitus did not correlate with the prognosis of facial nerve paralysis, suggesting that nerve damage caused by viral infection may have a more significant impact than these factors.

Other treatments

Intratympanic corticosteroid injection (ITSI) is utilized for the treatment of acute facial nerve paralysis. Chung et al. [27] reported no significant differences in the complete recovery rate between the conventional treatment group and the ITSI group (which received intratympanic dexamethasone three times every three to four days). However, patients in the ITSI group exhibited faster improvement in facial function within three weeks. Inagaki et al. [28] reported that the recovery rate was higher in the concurrent ITSI group (receiving intratympanic dexamethasone for 10 consecutive days) compared to the control group. Conversely, Kim et al. [29] reported no evidence supporting the benefit of ITSI. Thus, the efficacy of ITSI remains controversial.

Basic fibroblast growth factor (bFGF) has garnered significant attention for its application in various nerve injuries and tympanic membrane perforation [30,31]. Hato et al. [32] reported that, in cases of severe Bell’s palsy requiring facial nerve decompression surgery, the recovery rate was significantly better when a novel surgery, involving the placement of a bFGF-impregnated biodegradable gelatin hydrogel around the exposed nerve, was performed compared to conventional surgery. This result suggests that local administration of bFGF may also be effective.

Limitations

This study was retrospective rather than prospective, and the number of patients who received an initial dosage of 100 mg PSL was limited. Specifically, the comparison between the two groups with ENoG less than 10% was not possible due to the small number of patients, and a separate comparison within each disease for ENoG less than 20% was also not feasible for the same reason. Some patients did not receive confirmation of recovery or attend follow-up visits at our hospital for a duration of six months due to various reasons, including relocation, referral to other medical institutions, or voluntary discontinuation of care. As a result, the number of eligible patients was reduced. Furthermore, follow-up appointments were not consistently scheduled on a monthly basis because of either physician discretion or patient-initiated cancellations. Therefore, facial score assessments could not be conducted in certain months. Consequently, the results from the first to fifth months were obtained only from patients who visited during those points, leading to inconsistencies in the values over time. A larger sample size is necessary for a statistically robust analysis. Finally, prospective randomized controlled trials are essential to determine the optimal corticosteroid dosage for the treatment of Bell’s palsy and Ramsay Hunt syndrome.

## Conclusions

We retrospectively investigated the difference in therapeutic efficacy between standard and reduced HDCT groups in patients with Bell’s palsy and Ramsay Hunt syndrome. Although the reduced HDCT group was older and had a higher prevalence of hypertension and diabetes mellitus, the outcome of the therapeutic efficacy of the standard HDCT was not significantly higher, compared to the reduced HDCT. There were no significant differences in efficacy between the two regimens, suggesting that an initial dosage of more than 100 mg/day PSL with a taper does not enhance therapeutic outcomes for Bell’s palsy and Ramsay Hunt syndrome. Prospective randomized controlled trials are needed to further explore and confirm these findings.
